# A Comprehensive Characterization of Mitochondrial Genome in Papillary Thyroid Cancer

**DOI:** 10.3390/ijms17101594

**Published:** 2016-10-10

**Authors:** Xingyun Su, Weibin Wang, Guodong Ruan, Min Liang, Jing Zheng, Ye Chen, Huiling Wu, Thomas J. Fahey, Minxin Guan, Lisong Teng

**Affiliations:** 1 Department of Surgical Oncology, First Affiliated Hospital, School of Medicine, Zhejiang University, Hangzhou 310003, China; luckymaimai@sina.cn (X.S.); wbwang@zju.edu.cn (W.W.); 2 Department of Oncology, the Second Hospital of Shaoxing, Shaoxing 312000, China; recardos@163.com; 3 Institute of Genetics, School of Medicine, Zhejiang University, Hangzhou 310058, China; liangmin85685@126.com (M.L.); candy88zj@zju.edu.cn (J.Z.); yechency@zju.edu.cn (Y.C.); 4 Department of Plastic Surgery, First Affiliated Hospital, School of Medicine, Zhejiang University, Hangzhou 310003, China; whl1616@126.com; 5 Department of Surgery, New York Presbyterian Hospital and Weill Medical College of Cornell University, New York, NY 10021, USA; tjfahey@med.cornell.edu

**Keywords:** mitochondrial DNA, mitochondrial DNA copy number, haplogroup, papillary thyroid cancer

## Abstract

Nuclear genetic alterations have been widely investigated in papillary thyroid cancer (PTC), however, the characteristics of the mitochondrial genome remain uncertain. We sequenced the entire mitochondrial genome of 66 PTCs, 16 normal thyroid tissues and 376 blood samples of healthy individuals. There were 2508 variations (543 sites) detected in PTCs, among which 33 variations were novel. Nearly half of the PTCs (31/66) had heteroplasmic variations. Among the 31 PTCs, 28 specimens harbored a total of 52 somatic mutations distributed in 44 sites. Thirty-three variations including seven nonsense, 11 frameshift and 15 non-synonymous variations selected by bioinformatic software were regarded as pathogenic. These 33 pathogenic mutations were associated with older age (*p* = 0.0176) and advanced tumor stage (*p* = 0.0218). In addition, they tended to be novel (*p* = 0.0003), heteroplasmic (*p* = 0.0343) and somatic (*p* = 0.0018). The mtDNA copy number increased in more than two-third (46/66) of PTCs, and the average content in tumors was nearly four times higher than that in adjacent normal tissues (*p* < 0.0001). Three sub-haplogroups of N (A4, B4a and B4g) and eight single-nucleotide polymorphisms (mtSNPs) (A16164G, C16266T, G5460A, T6680C, G9123A, A14587G, T16362C, and G709A) were associated with the occurrence of PTC. Here we report a comprehensive characterization of the mitochondrial genome and demonstrate its significance in pathogenesis and progression of PTC. This can help to clarify the molecular mechanisms underlying PTC and offer potential biomarkers or therapeutic targets for future clinical practice.

## 1. Introduction

Mitochondria are semiautonomous organelles responsible for bioenergetic metabolism, aging and apoptosis [1]. Otto Warburg et al. first proposed that metabolic reprogramming occurred in cancer cells evidenced by highly activated glycolysis even in the presence of oxygen, and this was regarded as a hallmark of cancer [2]. This phenomenon, called the Warburg effect, is probably triggered by insufficient energy supply that is the result of the combination of mitochondrial defects and activated cellular proliferation [3]. Mitochondrial DNA (mtDNA) is a 16,569 bp, double-stranded circular molecule encoding 13 polypeptides, two ribosomal RNAs (rRNAs) and 22 transfer RNAs (tRNAs) for mitochondrial respiration. The replicative origins and transcriptive promoters are located in the non-coding displacement-loop (D-loop) region [4]. Accumulated evidence demonstrates that mtDNA variations and copy number alterations are common in human cancers [5]. Pathogenic mtDNA mutations can severely affect mitochondrial respiration and overproduce endogenous reactive oxygen species (ROS) contributing to anti-apoptosis, proliferation and metastasis of cancer [5,6].

Papillary thyroid cancer (PTC) is the main histological type of thyroid cancer. Most PTC patients have favorable outcome with the 30-year survival rate more than 90% after routine treatment by thyroidectomy with or without radioiodine ablation [7]. However, a small group of PTC patients suffer from tumor persistence, recurrence and even death [8]. Investigating the underlying molecular mechanisms of PTC can provide promising biomarkers and therapeutic targets for early diagnosis and treatment, thus improving prognosis and survival quality of patients, especially those with aggressive tumor behavior and adverse outcomes. The malignant transformation and progression of thyroid cancer is driven by accumulated genetic alterations. Among them, the BRAF^V600E^ mutation is the most significant factor for PTC and is associated with high-risk clinicopathological features and unfavorable outcomes [9]. Therefore, many researchers suggest that BRAF^V600E^ mutation can be a valuable biomarker and therapeutic target for diagnosis, risk stratification, prognostic prediction and treatment of PTC [9].

In spite of the research achievements in understanding the nuclear genome, the role of mitochondrial genome in pathogenesis and progression of thyroid cancer is still incompletely characterized. Previous researchers have found abnormally excessive mitochondria and prevalent mtDNA alterations in thyroid cancer. However, the majority of these studies are restricted to the oncocytic subtype of thyroid cancer and only focused on mutation hotspots of mtDNA [10,11,12]. Here we comprehensively characterized the mitochondrial genome in papillary thyroid cancer by sequencing the entire mtDNA of 66 PTCs, 16 normal thyroid tissues and 376 blood samples of healthy individuals. The mtDNA variation distribution, haplogroup and copy number were further analyzed.

## 2. Results

### 2.1. Distribution of mtDNA Variations

A total of 2508 variations in 543 sites were identified in 66 PTC cases, and the D-loop region was the hotspot of mtDNA (Figure S1a,b). Single-base substitution was the main component of mtDNA variations, in addition to 76 deletions (13 sites) and 112 insertions (10 sites) (Figure S1c). About 30.9% (101/327) transitions and 60% (12/20) transversions were non-synonymous, suggesting that transversion was more likely to alter the encoded amino-acid and affect the structure or function of protein (Figure S1d). In the protein-coding region, most variations were synonymous (Figure S1e). ATPase6 (14/22, 63.6%), Cytb (20/45, 44.4%), ND4L (3/8, 37.5%) and ND5 (25/71, 35.2%) genes harbored relatively high ratio of nonsynonymous variation (Figure S1e,f). A total of 33 variations—including 11 non-synonymous, seven nonsense and eight frameshift variations—in 25 PTC patients were novel, and all of them were singular (Table 1). Heteroplasmy was one of the most important characteristics of mitochondrial genome, presenting in nearly half of the 66 PTCs (31/66). Among the heteroplasmic variations, 52 somatic mutations (44 sites) in 28 PTC patients and 28 germline variations (20 sites) in 16 patients were detected (Table S1).

### 2.2. The mtDNA Variations in Non-Coding Region

**Figure 1:**
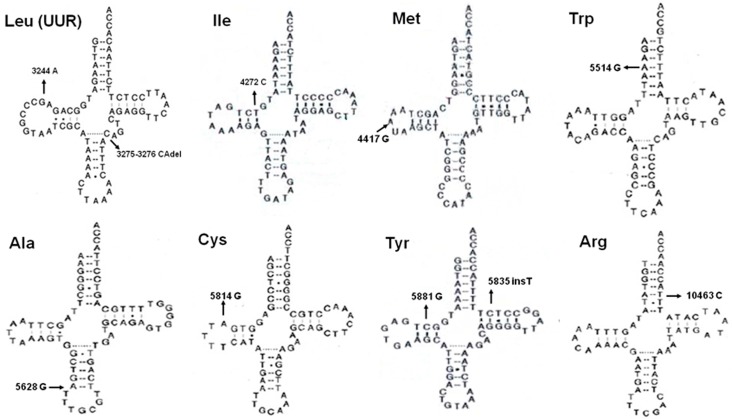
Potential pathogenic tRNA variations in PTC Schematic structures of eight mitochondrial tRNAs are shown. Arrows point out the position of tRNA variation.

There were 103 substitutions and 10 frameshift alterations in D-loop region. Nearly all the insertions and deletions were located in mitochondrial microsatellite instability (mtMSI) regions, such as poly-C in np 303–315 or np 16184–16193 and poly-CA stretch in np 514–523. In the RNA region, 20, 21 and 29 variations were, respectively, identified in 12S rRNA, 16S rRNA and tRNAs. The published secondary structures of RNAs were used to localize the alterations in the stem and loop structure [13]. A total of seven variations in 12S rRNA, one alteration in 16S rRNA and 13 alterations in tRNAs changed the Waston–Crick base-pairing. According to their frequencies in control groups and conservation of the altered nucleotides, 13 variations were identified as potentially deleterious and five of them had been reported in diseases (Table S2, Figure 1).

### 2.3. The mtDNA Variations in Protein-Coding Region

**Figure 2:**
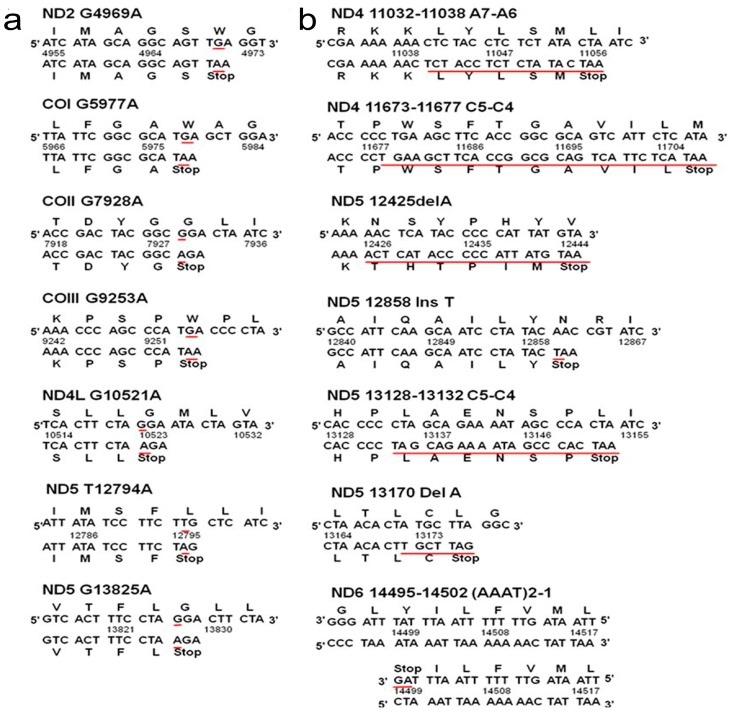
The nonsense and framshift mutations: (**a**) Seven nonsense mutations directly introduce stop-codon and thus create premature termination of protein synthesis immediately; and (**b**) Seven frameshift alterations bring stop-codon in the following transcription and induce truncated polypeptide.

A total of 234 synonymous, 113 non-synonymous, seven nonsense and 11 frameshift variations were detected in protein-coding region. All the nonsense and frameshift variations brought in advanced stop-codon (UAG, UGA) and leaded to premature termination of protein synthesis (Table 2, Figure 2). Among the 113 non-synonymous alterations, 26 variations were selected as potentially pathogenic based on their frequencies in control groups and conservation of the altered amino-acid (Table 3). These 26 selected variations were further evaluated by seven bioinformatic programs, and 15 of them were predicted as deleterious by more than half of the programs (Table 3). Therefore, these 33 mutations in 32 patients, including 15 nonsynonymous, seven nonsense and 11 frameshift mutations, were classified as pathogenic mutations. These pathogenic mtDNA mutations were associated with patients’ older age (*p* = 0.018) and advanced tumor stage (*p* = 0.022), and tended to be novel (*p* < 0.001), heteroplasmic (*p* = 0.034) and somatic (*p* = 0.002) (Table S3).

### 2.4. The Alteration of mtDNA Copy Number

**Figure 3:**
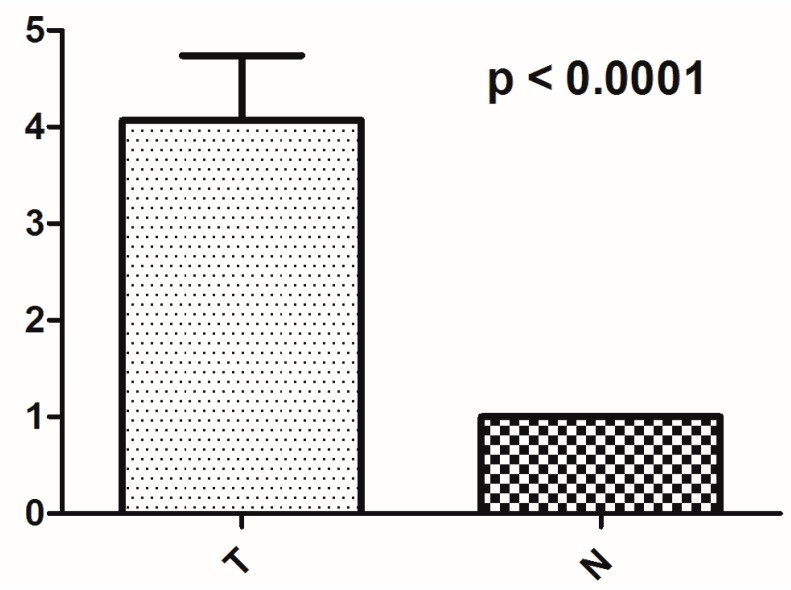
Copy number analysis of mtDNA in thyroid cancer: comparison of the average mtDNA copy number between PTC cases (T) and their corresponding normal tissues (N). Two-sided Mann–Whitney *U* test was used to analysis the difference, and *p* < 0.05 was considered as significant.

In comparison with corresponding normal tissues, more than two-thirds (46/66) of the PTCs had increased mtDNA copy number. The average mtDNA content in tumors was nearly four times higher than that in adjacent normal tissues (*p* < 0.0001) (Figure 3). Interestingly, mtDNA content in the tumor of patient No. 48 was more than 38 times higher than the corresponding normal tissue. However, our analysis showed that increased mtDNA content had no significant association with clinicopathological features. No obvious association was observed between mtDNA content with novel or heteroplasmic mtDNA variations, PTC-associated mutations or mtMSIs (309insC/CC and 523del/insCA).

### 2.5. Analysis of Haplogroup and mtSNP

**Figure 4:**
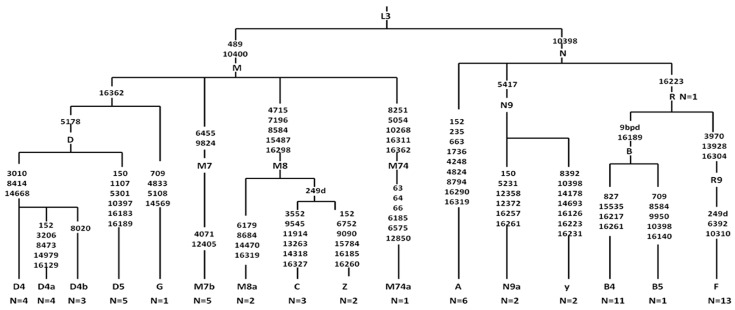
Phylogenetic tree was constructed to reveal the underlying lineages of 16 mtDNA haplogroups in 66 PTC cases.

The entire mtDNA sequences of 66 PTCs were assigned to Asian mtDNA lineage and classified into 11 haplogroups distributed between macro-haplogroups M (*n* = 30) and N (*n* = 36). Sub-haplogroups were descended from macro-haplogroups M (C, D, G and Z) and N (A, B, F, N, R and Y) (Figure 4). Although no statistical significance was found in haplogroup M or N, the sub-haplogroups A4 (OR 3.903, 95% CI 1.070–14.23, *p* = 0.027), B4a (OR 3.903, 95% CI 1.070–14.23, *p* = 0.027) and B4g (OR 11.5, 95% CI 1.027–128.8, *p* = 0.013) descending from haplogroup N tended to be associated with the occurrence of PTC (Table S4). Frequencies of 15 mtSNPs were statistically different between PTC and healthy groups, and eight of them (A16164G, T16362C, C16266T, G5460A, T6680C, G9123A, A14587G, and G709A) may be associated with a predisposition to developing PTC according to their frequencies between PTC and normal thyroid groups (Table S5).

## 3. Discussion

In spite of generally indolent behavior and favorable prognosis associated with papillary thyroid cancer, tumor recurrence and distant metastasis are intractable issues in the clinical treatment of a subset of PTC patients [8]. Identifying high-risk patients and offering appropriate, more aggressive therapy in the early stages has been an important goal for clinical researchers. Considering the crucial role of mitochondria in carcinogenesis, investigation of mitochondrial genome may provide potential biomarkers and therapeutic targets for clinical practice. Here we identified 33 pathogenic mtDNA mutations in the protein-coding region, and found three sub-haplogroups and eight mtSNPs that were associated with PTC predisposition. In addition, the average mtDNA copy number in PTCs was significantly higher than that in corresponding normal tissues.

The mutation load of mtDNA is 10–20 times higher than nuclear DNA, probably because the protect and repair system in mitochondria is insufficient and mtDNA is more vulnerable to oxidative stress generated by oxidative phosphorylation [14]. The D-loop region is a mutation hotspot of mtDNA due to the unique triple-stranded DNA structure [15]. The mtMSIs in the D-loop region can modify the binding affinity of transacting elements and direct the formation of persistent RNA-DNA hybrids regulating the efficiency of replication and transcription, which are probably produced by direct oxidative attack, slippage or mis-incorporation during replication and inefficient repair of polymerase. The mtDNA copy number varies in different cell types and microenvironments, and is precisely modulated by alterations in the D-loop region. The content of mtDNA is important for functional maintenance of mitochondria, but alterations in mtDNA and their significance in different types of cancer are still discrepant [16]. Our analysis found excessive replication of mtDNA in PTCs. However, no significant association was presented between mtDNA content and clinicopathological features, and no obvious association was observed between mtDNA content with novel or heteroplasmic mtDNA variations, PTC-associated mutations or mtMSIs. Probably, other factors also take part in the increased copy number of mtDNA. Corver et al. demonstrated that the presence of near-homozygous genome (NHG), rather than damaging or disruptive mtDNA mutations, was correlated with oncocytic phenotype which showed a strikingly mitochondrial proliferation [10]. Interestingly, mtDNA content in tumor of No. 48, a conventional variant PTC, was more than 38 times higher than corresponding normal tissue. In this specimen, we identified a novel frameshift alteration 14495–14502 del (AAAT) in the ND6 gene, which directly resulted in a premature stop-codon (UAG) being introduced and truncated the polypeptide from 175 amino-acid to 58 amino-acid. Thus we speculate that the highly increased mtDNA copy number may have been triggered by defective mitochondrial function caused by this novel deletion [10,16].

Heteroplasmy is a unique characteristic of mitochondrial genome, and also a typical feature of pathogenicity [17]. Once the pathogenic threshold is surpassed, the heteroplasmic level can affect the biochemical and clinical phenotype from mild functional deficiency to complete disassembly of the mitochondrial complex [18]. In our study, nearly half of the PTC cases harbored heteroplasmic variations. Among these heteroplasmic variations, 52 variations were somatic and the majority of them were novel—which dramatically increases their likelihood of being cancer-specific [19]. These somatic variations may confer a neoplastic advantage for tumor cells, and their successive introduction within a developing tumor may provide necessary genetic diversity to satisfy the adaptive evolution and drive tumor progression [20].

Cybrid models have demonstrated that the mitochondrion, but not nuclei, is the master contributor to mitochondrial dysfunction [21]. Pathogenic mtDNA mutations can hamper the electron transport chain (ETC) and generate excessive electrons, which triggers cancer-associated pathways and in turn produces more mutations aggravating the respiratory deficiency. It is reported that more than half of the pathogenic mutations are located in tRNAs which comprise only 10% coding capacity of mitochondrial genome, while the protein-coding region occupying about 70% mtDNA accounts for 40% disease-related mutations. The two rRNAs harbor only about 2% of the pathogenic mutations [20]. In the RNA genes, we identified 13 possibly detrimental variations and five of them had been previously reported in diseases according to the Mitomap database. For example, G3244A in tRNA^Leu(UUR)^, next to the famous pathogenic mutation A3243G, was first detected in mitochondrial myopathy, encephalopathy, lactic acidosis, and stroke-like episodes (MELAS) and later found in several cancers including oncocytic thyroid tumors [22,23]. The A5514G in tRNA^Trp^ damaging an A–U base-pair in ACC-stem was identified in neonatal onset mito-disease and analyzed to be damaging by clinicopathology and biochemistry [24]. The T5628C in tRNA^Ala^ disrupted an extremely conserved A–U base-pair in the anti-codon stem and resulted in nine unmatched nucleotides (rather than the seven in normal cells) which decreased the energetic stability of tRNA^Ala^ [25].

In the coding region, seven nonsense and 11 frameshift mutations introduced premature stop-codons (UAG, UGA) in protein synthesis and resulted in loss-of-function or even disassembly of the complex. Among them, both 10952insC and 11032–11038delA have been detected in renal oncocytoma [26], and 11032-11038delA was also found in prostate cancer [27]. The 12425delA has been previously identified in a girl having chronic renal failure, persistent lactic acidosis and myopathy [28]. A similar variation 12425insA has been reported in several cancers in a heteroplasmic status [29,30]. These nonsense and frameshift mutations, together with 15 non-synonymous mutations selected by bioinformatics programs, were regarded as pathogenic, and may interfere the OXPHOS system of mitochondrial respiration and contribute to the molecular pathogenesis of thyroid cancer. The association between these pathogenic mtDNA mutations and advanced tumor stage suggests the possible involvement of mtDNA mutations in malignant transformation and progression. Apart from pathogenic mutations, “non-pathologic” mtSNPs can also affect carcinogenesis and progression of cancer in multifactorial manners. Haplotypes, classified by specific combinations of tightly linked mtSNPs, are also correlated with the predisposition to specific cancers [31]. For example, haplogroup U increased the risk of prostate cancer and renal cancer in white North American individuals [32], but decreased the risk of breast cancer in European-American women [33].

The application value of mtDNA variations in early diagnosis, risk stratification, prognostic prediction and disease monitoring of cancer have been widely investigated and discussed. Since mtDNA is a small size and close-circular molecular entity and does not undergo recombination, mtDNA variations are more fixed and persistent than nuclear alterations. Due to the high copy number of mtDNA, detecting mtDNA biomarkers can be more sensitive and powerful than nuclear ones. Therefore, mtDNA biomarkers may have special advantage in samples of limited cellularity including fine-needle aspiration or core-needle aspiration of thyroid nodule. Furthermore, mitochondria are potential therapeutic targets for cancer treatment and can be specifically targeted by antioxidant compounds, selective gene-therapy or approaches changing the mtDNA variation load. Dai et al. demonstrated that mitophagy induced by rapamycin can eliminate pathogenic mtDNA mutations and increase ATP restoration [34]. Recently, several researchers reported that resistance to BRAF inhibition was partly caused by increased mitochondrial biogenesis and oxidative respiration, and therapies inhibiting this metabolic reprogramming restored the function of the BRAF inhibitor and improved treatment efficiency [35,36].

The major limitation of our study is that we do not analyze the mitochondrial genome in anaplastic thyroid cancer (ATC), which has more aggressive behaviors and worse prognosis than PTC. We plan to analyze the mitochondrial characteristics in ATC and evaluate their clinical and prognostic significance. Furthermore, we can compare the role of mitochondrial genome in different histological types of thyroid cancer.

## 4. Materials and Methods

### 4.1. Sample Collection

A total of 66 PTC patients underwent primary surgery in the First Affiliated Hospital, Zhejiang University School of Medicine (Hangzhou, China) were enrolled. None of them had a history of cancer or radiotherapy before surgery. Histopathology of tumor specimens was independently evaluated by two experienced pathologists according to the World Health Organization (WHO) classification [37]. Among the 66 PTCs, two were follicular variant and the others were classical variant. Tumors and adjacent normal tissues were immediately frozen in −80 °C after resection. The 16 normal thyroid tissues were used to distinguish tissue-specific variations, and 376 blood samples of healthy individuals from the same geographic region were collected to identify polymorphisms in this population. All the samples were obtained with informed consent. The study was conducted in accordance with the Declaration of Helsinki, and the protocol was approved by the Ethics Committee of the First Affiliated Hospital, College of Medicine, Zhejiang University (2015-443, 30 December 2015).

### 4.2. Sequencing of the Mitochondrial Genome

Genomic DNA was isolated from frozen tissues and blood samples using a commercial kit (QIAamp DNA Mini Kit from QIAGEN, Hilden, Germany). Concentration and purity of DNA were analyzed by spectrometry. The entire mitochondrial genome was PCR-amplified by 24 pairs of overlapping primer as described previously [38]. The PCR products were detected by electrophoresis in 1% agarose gel and then sequencing by the ABI 3700 automated DNA sequencer (Applied BioSystems, Foster City, CA, USA) using BigDye Terminator v3.1 Cycle Sequencing Kit (Applied BioSystems).

### 4.3. Sequence Analysis and Haplogroup Classification

The sequences of mtDNA were aligned to the revised Cambridge Reference Sequence (rCRS) (GeneBank accession number: NC_012920) to identify mtDNA variations [39]. The variation load referred to the percentage of variations per gene or complex, which was calculated as follows: total number of altered nucleotides per gene or complex/total number of nucleotides per gene or complex ×100. Variations not recorded in the Mitomap database (http://www.mitomap.org) were regarded as novel. All the heteroplasmic variations were confirmed by repeat analysis of the other strand and compared with the corresponding positions in adjacent normal tissues. The mtDNA haplogroups were classified according to the updated phylogenetic tree of mtDNA (mtDNA tree Built 16) provided by PhyloTree (http://www.phylotree.org) [31].

### 4.4. Phylogenetic Conservation Analysis and Pathogenic Prediction

Inter-species conservation of the altered amino acids or nucleotides was evaluated by mitochondrial sequences of 41 primates (Table S6). The conservation index (CI) was defined as the percentage of species having wild-type amino-acid or nucleotide by comparing the amino-acid or nucleotide of human with the other 40 species. The higher the conservation of the altered amino-acid or nucleotide was, the greater the pathogenic possibility will be. The variations with potential pathogenicity were selected based on the following criteria: (1) presented in less than 1% of 376 healthy individuals—those variations existed in more than 1% healthy controls were regarded as polymorphisms; (2) were absent in normal thyroid samples, and those variations also identified in normal thyroid samples were regarded as tissue-specific variations; and (3) the altered amino-acids or nucleotides had high conservation (CI > 75%), which indicated the high possibility of functional consequence. Furthermore, the potentially pathogenic variations in protein-coding region were evaluated by 7 bioinformatic programs including PolyPhen-2 (http://genetics.bwh.harvard.edu/pph2/), SIFT (http://sift.jcvi.org/), MutationAssessor (http://mutationassessor.org/), Provean (http://provean.jcvi.org/index.php), SNP & GO (http://snps-and-go.biocomp.unibo.it/), Align GVGD (https://www.biostars.org/) and PANTHER (http://fathmm.biocompute.org.uk/). The variations that were predicted as deleterious by more than half of these 7 programs had high possibility to be “pathogenic” for mitochondrial function and associated with PTC.

### 4.5. Determination of mtDNA Copy Number

The mtDNA content relative to nuclear encoded 18s RNA was determined by quantitative real-time PCR in ABI Prim 7900HT system using FastStart Universal SYBR Green Master Mix (Roche Diagnostics GmbH, Mannheim, Germany). The primers used for amplification of mtDNA copy number were: the forward primer 5′ CACCCAAGAACAGGGTTTGT 3′ and the reverse primer 5′ TGGCCATGGGTATGTTGTTAA 3′. Another pair of primers was designed to amplify 18s RNA: the forward primer 5′ TAGAGGGACAAGTGGCGTTC 3′ and the reverse primer 5′ CGCTGAGCCAGTCAGTGT 3′. The total volume of PCR mixture was 10 μL including 2 μL DNA (2 ng/μL), 3 μL primers (10 μM) and 5 μL SYBR Green Master Mix. The action was conducted as follows: 50 °C for 2 min, 95 °C for 10 min and followed by 45 cycles of 95 °C for 5 s, 58 °C for 30 s and 72 °C for 1 min. All the reactions were repeated 3 times. Non-template control and a serial dilution of reference DNA were used in each reaction.

### 4.6. Statistical Analysis

All the statistical analyses were conducted by SPSS software (version 21.0) (SPSS Inc., Chicago, IL, USA). The Pearson chi-square test was performed to analyze the clinicopathological significance of mitochondrial characteristics. Two-sided Mann–Whitney *U* test was used to analyze the difference of the average mtDNA copy number between PTC cases and their corresponding normal tissues. The odds ratios (ORs) with 95% confidence intervals (CIs) were calculated to clarify the association of haplogroups and single-nucleotide polymorphisms (mtSNPs) with PTC occurrence. For all analyses, *p* < 0.05 was regarded as statistically significant.

## 5. Conclusions

Here, we have reported a comprehensive characterization of the mitochondrial genome in PTC, and demonstrated that pathogenic mtDNA mutations, as well as some specific mtSNPs and haplogroups, may be involved in the pathogenesis and progression of PTC. These results provide an alternative dimension to clarify the molecular mechanisms underlying PTC carcinogenesis, and present possible novel biomarkers and therapeutic targets for the diagnosis, risk stratification, prognostic prediction and treatment of papillary thyroid cancer.

## Figures and Tables

**Table 1:** Novel mtDNA variations in the entire mitochondrial genome.

Position	Gene	Replacement	Amino-Acid Change or Watson-Crick Base-Pairing ^a^	Conservation Index (%) ^b^	Number of 66 PTC Patients (%)	Number of 376 Healthy Controls (%)	Heter/Homo ^c^
RNA Region							
1629	tRNA^Val^	A–T	A–U↓	24.4%	1 (1.52%)	0 (0.00%)	Homo
2274	16S rRNA	A–G		100%	1 (1.52%)	0 (0.00%)	Heter
3275–3276	tRNA^Leu(UUR)^	Del CA		-	1 (1.52%)	0 (0.00%)	Heter
4272	tRNA^Ile^	T–C	A–U↓	100%	1 (1.52%)	0 (0.00%)	Homo
5835	tRNA^Tyr^	Ins T		-	1 (1.52%)	0 (0.00%)	Homo
5881	tRNA^Tyr^	G–C	C–G↓	100%	1 (1.52%)	0 (0.00%)	Homo
10040	tRNA^Gly^	C–A		43.9%	1 (1.52%)	0 (0.00%)	Homo
Protein-Coding Region							
4520–4521	ND2	Del AC	-	-	1 (1.52%)	0 (0.00%)	Homo
4875	ND2	C–T	Leu -> Leu	100%	1 (1.52%)	0 (0.00%)	Homo
4969	ND2	G–A	No: Trp -> Ter ^d^	100%	1 (1.52%)	0 (0.00%)	Homo
4971	ND2	G–A	No: Gly -> Ser	100%	1 (1.52%)	0 (0.00%)	Homo
5977	COI	G–A	No: Trp -> Ter	100%	1 (1.52%)	0 (0.00%)	Heter
6238	COI	T–C	No: Leu -> Pro	100%	1 (1.52%)	0 (0.00%)	Heter
7104	COI	T–C	No: Ser -> Pro	100%	1 (1.52%)	0 (0.00%)	Heter
7750	COII	C–A	No: Ile -> Met	58.5%	1 (1.52%)	0 (0.00%)	Homo
7928	COII	G–A	No: Gly -> Ter	56.1%	1 (1.52%)	0 (0.00%)	Homo
9253	COIII	G–A	No: Trp -> Ter	100%	1 (1.52%)	0 (0.00%)	Heter
10521	ND4L	G–A	No: Gly -> Ter	100%	1 (1.52%)	0 (0.00%)	Homo
10622	ND4L	C–T	Thr -> Thr	36.6%	1 (1.52%)	0 (0.00%)	Homo
11646	ND4	Ins T	-	-	1 (1.52%)	0 (0.00%)	Homo
11673–11677	ND4	C5–C4	-	-	1 (1.52%)	0 (0.00%)	Heter
11673–11677	ND4	C5–C6	-	-	1 (1.52%)	0 (0.00%)	Homo
12794	ND5	T–A	No: Leu -> Ter	100%	1 (1.52%)	0 (0.00%)	Heter
12858	ND5	Ins T	-	-	1 (1.52%)	0 (0.00%)	Heter
12943	ND5	C–T	No: Leu -> Phe	24.4%	1 (1.52%)	0 (0.00%)	Heter
13128–13132	ND5	C5–4	-	-	1 (1.52%)	0 (0.00%)	Homo
13170	ND5	Del A	-	-	1 (1.52%)	0 (0.00%)	Homo
13621	ND5	C–T	No: Leu -> Phe	51.2%	1 (1.52%)	0 (0.00%)	Homo
13825	ND5	G–A	No: Gly -> Ter	100%	1 (1.52%)	0 (0.00%)	Homo
14310	ND6	C–A	No: Gly -> Trp	70.7%	1 (1.52%)	0 (0.00%)	Heter
14495–14502	ND6	(AAAT)2–1	-	-	1 (1.52%)	0 (0.00%)	Homo
14774	Cytb	C–A	No: Leu -> Ile	63.4%	1 (1.52%)	0 (0.00%)	Heter
15018	Cytb	T–A	No: Phe -> Tyr	100%	1 (1.52%)	0 (0.00%)	Heter

^a^ Watson–Crick base-pairing: abolished (↓); ^b^ Conservation index denotes the conservative properties of amino-acid or nucleotides in 41 primate species; ^c^ Heter: Heteroplasmy; Homo: Homoplasmy; ^d^ Ter: Terminator.

**Table 2:** Nonsense and frameshift mutations identified in protein-coding region.

Position	Gene	Change	Reported ^a^	Number of 66 PTC Patients (%)	Number of 16 Normal Thyroid Tissues (%)	Number of 376 Healthy Controls (%)	Heter/Homo ^b^
**Nonsense Mutation**							
4969	ND2	G–A	N	1 (1.52%)	0 (0.00%)	0 (0.00%)	Homo
5977	COI	G–A	N	1 (1.52%)	0 (0.00%)	0 (0.00%)	Heter
7928	COII	G–A	N	1 (1.52%)	0 (0.00%)	0 (0.00%)	Homo
9253	COIII	G–A	N	1 (1.52%)	0 (0.00%)	0 (0.00%)	Heter
10521	ND4L	G–A	N	1 (1.52%)	0 (0.00%)	0 (0.00%)	Homo
12794	ND5	T–A	N	1 (1.52%)	0 (0.00%)	0 (0.00%)	Heter
13825	ND5	G–A	N	1 (1.52%)	0 (0.00%)	0 (0.00%)	Homo
**Frameshift Mutation**							
4520–4521	ND2	Del AC	N	1 (1.52%)	0 (0.00%)	0 (0.00%)	Homo
10952	ND4	Ins C	Y	1 (1.52%)	0 (0.00%)	0 (0.00%)	Homo
11032–11038	ND4	A7–6	Y	4 (6.06%)	0 (0.00%)	0 (0.00%)	Homo + Heter
11646	ND4	Ins T	N	1 (1.52%)	0 (0.00%)	0 (0.00%)	Homo
11673–11677	ND4	C5–C4	N	1 (1.52%)	0 (0.00%)	0 (0.00%)	Heter
11673–11677	ND4	C5–C6	N	1 (1.52%)	0 (0.00%)	0 (0.00%)	Homo
12418–12425	ND5	Del A	Y	1 (1.52%)	0 (0.00%)	0 (0.00%)	Heter
12858	ND5	Ins T	N	1 (1.52%)	0 (0.00%)	0 (0.00%)	Heter
13128–13132	ND5	C5–4	N	1 (1.52%)	0 (0.00%)	0 (0.00%)	Homo
13170	ND5	Del A	N	1 (1.52%)	0 (0.00%)	0 (0.00%)	Homo
14495–14502	ND6	(AAAT)2–1	N	1 (1.52%)	0 (0.00%)	0 (0.00%)	Homo

^a^ According to Mitomap (http://www.mitomap.org); ^b^ Heter: Heteroplasmy; Homo: Homoplasmy.

**Table 3:** Potential pathogenic mtDNA variations identified in protein-coding region.

Position	Gene	Change	Amino-Acid Change	Conservation Index (%) ^a^	Reported ^b^	Number of 66 PTC Patients (%)	Number of 16 Normal Thyroid Tissues (%)	Number of 376 Healthy Controls (%)	Polyphen-2 ^c^	SIFT	Mutation Assesor	Provean	SNP&GO	Align GVGD ^d^	PANTHER (Pdeleterious) ^e^
*3392 ^f^*	ND1	G–A	No: Gly -> Asp	100.00%	Y	1 (1.52%)	0 (0.00%)	0 (0.00%)	Probably	Not Tolerated	High	Deleterious	Disease	C65	NA ^g^
*3644*	ND1	T–C	No: Val -> Ala	97.60%	Y	1 (1.52%)	0 (0.00%)	2 (0.53%)	Benign	Not Tolerated	Medium	Deleterious	Neutral	C65	0.29125
*3679*	ND1	T–C	No: Ser -> Pro	100.00%	Y	1 (1.52%)	0 (0.00%)	0 (0.00%)	Probably	Not Tolerated	High	Deleterious	Disease	C65	0.74261
3745	ND1	G–A	No: Ala -> Thr	92.70%	Y	1 (1.52%)	0 (0.00%)	0 (0.00%)	Benign	Not Tolerated	Low	Neutral	Neutral	C55	0.21113
*4971*	ND2	G–A	No: Gly -> Ser	100.00%	N	1 (1.52%)	0 (0.00%)	0 (0.00%)	Probably	Not Tolerated	Medium	Deleterious	Neutral	C55	0.36251
*6238*	COI	T–C	No: Leu -> Pro	100.00%	N	1 (1.52%)	0 (0.00%)	0 (0.00%)	Probably	Not Tolerated	High	Deleterious	Disease	C65	0.87509
6340	COI	C–T	No: Thr -> Ile	82.90%	Y	1 (1.52%)	0 (0.00%)	0 (0.00%)	Benign	Not Tolerated	Medium	Neutral	Neutral	C65	0.21096
6681	COI	T–C	No: Tyr -> His	85.40%	Y	1 (1.52%)	0 (0.00%)	0 (0.00%)	Benign	Tolerated	Neutral	Neutral	Neutral	C65	0.32881
*7104*	COI	T–C	No: Ser -> Pro	100.00%	N	1 (1.52%)	0 (0.00%)	0 (0.00%)	Possibly	Not Tolerated	Neutral	Neutral	Disease	C65	0.5134
7329	COI	T–C	No: Phe ->Leu	100.00%	N	1 (1.52%)	0 (0.00%)	0 (0.00%)	Benign	Tolerated	Low	Neutral	Neutral	C15	0.16379
*8156*	COII	G–A	No: Val -> Met	75.61%	N	1 (1.52%)	0 (0.00%)	0 (0.00%)	Probably	Not Tolerated	Medium	Neutral	Neutral	C15	0.53442
*8989*	ATP6	G–A	No: Ala -> Thr	100.00%	Y	1 (1.52%)	0 (0.00%)	0 (0.00%)	Probably	Not Tolerated	Low	Deleterious	Neutral	C55	0.47286
*9187*	ATP6	T–C	No: Tyr -> His	100.00%	Y	1 (1.52%)	0 (0.00%)	0 (0.00%)	Probably	Not Tolerated	High	Deleterious	Disease	C65	NA
9355	COIII	A–G	No: Asn -> Ser	82.90%	Y	1 (1.52%)	0 (0.00%)	0 (0.00%)	Benign	Tolerated	Neutral	Neutral	Neutral	C45	0.14014
*10573*	ND4L	G–A	No: Gly -> Glu	97.60%	Y	1 (1.52%)	0 (0.00%)	0 (0.00%)	Probably	Not Tolerated	High	Deleterious	Neutral	C65	0.40946
12850	ND5	A–G	No: Ile -> Val	90.20%	Y	1 (1.52%)	0 (0.00%)	0 (0.00%)	Possibly	Tolerated	Neutral	Neutral	Neutral	C25	0.50297
13535	ND5	A–G	No: Asn -> Ser	87.80%	Y	1 (1.52%)	0 (0.00%)	0 (0.00%)	Benign	Not Tolerated	Low	Deleterious	Neutral	C45	NA
13748	ND5	A–G	No: Asn -> Ser	85.40%	Y	1 (1.52%)	0 (0.00%)	0 (0.00%)	Benign	Tolerated	Neutral	Neutral	Neutral	C45	0.5082
*14310*	ND6	C–A	No: Gly -> Trp	78.05%	N	1 (1.52%)	0 (0.00%)	0 (0.00%)	Probably	Not Tolerated	Medium	Deleterious	Disease	C65	0.71527
14463	ND6	T–C	No: Thr -> Ala	90.20%	Y	1 (1.52%)	0 (0.00%)	0 (0.00%)	Benign	Tolerated	Neutral	Deleterious	Neutral	C55	0.15283
*15018*	Cytb	T–A	No: Phe -> Tyr	100.00%	N	1 (1.52%)	0 (0.00%)	0 (0.00%)	Possibly	Not Tolerated	High	Deleterious	Disease	C15	0.68543
*15045*	Cytb	G–A	No: Arg -> Gln	100.00%	Y	1 (1.52%)	0 (0.00%)	0 (0.00%)	Probably	Not Tolerated	High	Deleterious	Disease	C35	0.59378
*15090*	Cytb	T–C	No: Ile -> Thr	85.40%	Y	1 (1.52%)	0 (0.00%)	1 (0.27%)	Possibly	Tolerated	Low	Deleterious	Neutral	C65	0.42865
15479	Cytb	T–C	No: Phe -> Leu	80.50%	Y	1 (1.52%)	0 (0.00%)	0 (0.00%)	Benign	Tolerated	Low	Deleterious	Neutral	C15	0.39962
*15483*	Cytb	C–T	No: Ser -> Leu	80.50%	Y	1 (1.52%)	0 (0.00%)	0 (0.00%)	Possibly	Tolerated	Low	Deleterious	Neutral	C65	0.45816

^a^ Conservation index denotes the conservative properties of amino-acid or nucleotides in 41 primate species; ^b^ According to Mitomap (http://www.mitomap.org); ^c^ Polyphen-2 classified the variations as probably damaging, possibly damaging and benign according to their pathogenic potential; ^d^ Align GVGD classified the variations as C65, C55, C45, C35, C25, C15 and C0 according to the risk estimates, and here we regarded the C65 as pathogenic; ^e^ PANTHER predicted the pathogenicity of variations by values of Pdeleterious, and we regarded Pdeleterious >0.5 as deleterious; ^f^ The variants predicted as by more than half of the bioinformatic software packagess were classified as PTC-associated mutations which were highlighted by bold and italic; ^g^ NA, not available.

## Supplementary Materials

Supplementary Materials can be found at www.mdpi.com/1422-0067/17/10/1594/s1.
