# Supplementary material for: A Comprehensive Characterization of Mitochondrial Genome in Papillary Thyroid Cancer
Source: Int J Mol Sci. 2016 Oct 10;17(10):1594. doi: 10.3390/ijms17101594 (PMC5085627; doi:10.3390/ijms17101594)
Supplement: Supplementary file 1 [file ijms-17-01594-s001.pdf]

Supplementary Materials: A Comprehensive Characterization of Mitochondrial Genome in Papillary Thyroid Cancer

Xingyun Su, Weibin Wang, Guodong Ruan, Min Liang, Jing Zheng, Ye Chen, Huiling Wu, Thomas J. Fahey III, Minxin Guan and Lisong Teng

Table S1. Heteroplasmic mtDNA variations in entire mitochondrial genome.

Position	Gene	Change	Amino-Acid Change or Watson-Crick Base-Pairing	Conservation Index (%) [a]	Reported [b]	Number of 66 PTC Patients (%)	Number of 376 Healthy Controls (%)	Somatic/Germline
**RNA region**								
2274	16S rRNA	A–G	–	100%	N	1 (1.52%)	0 (0.00%)	Somatic
3183	16S rRNA	T–C	–	80.5%	N	1 (1.52%)	0 (0.00%)	Somatic
3275–3276	$tRNA^{Leu (UUR)}$	Del CA	–	-	N	1 (1.52%)	0 (0.00%)	Somatic
14675	$tRNA^{Glu}$	A–G	–	61%	Y	2 (3.03%)	0 (0.00%)	Somatic
14693	$tRNA^{Glu}$	A–G	–	97.6%	Y	2 (3.03%)	6 (1.6%)	Somatic
**Protein-coding region**								
3392	ND1	G–A	No: Gly -> Asp	100%	Y	1 (1.52%)	0 (0.00%)	Somatic
5977	COI	G–A	No: Trp -> Ter [c]	100%	N	1 (1.52%)	0 (0.00%)	Somatic
6238	COI	T–C	No: Leu -> Pro	100%	N	1 (1.52%)	0 (0.00%)	Somatic
6596	COI	C–T	Tyr -> Tyr	100%	Y	1 (1.52%)	0 (0.00%)	Germline
7104	COI	T–C	No: Ser -> Pro	100%	N	1 (1.52%)	0 (0.00%)	Somatic
7822	COII	A–G	Pro -> Pro	100%	Y	1 (1.52%)	3 (0.8%)	Germline
8005	COII	T–C	Asn -> Asn	97.6%	Y	1 (1.52%)	0 (0.00%)	Germline
8156	COII	G–A	No: Val -> Met	75.61%	Y	1 (1.52%)	0 (0.00%)	Somatic
9253	COIII	G–A	No: Trp -> Ter	100%	N	1 (1.52%)	0 (0.00%)	Somatic
10573	ND4L	G–A	No: Gly -> Glu	100%	Y	1 (1.52%)	0 (0.00%)	Somatic
11032–11038	ND4	A7–6	-	-	Y	4 (6.1%)	0 (0.00%)	Somatic
11673–11677	ND4	C5–C4	-	-	N	1 (1.52%)	0 (0.00%)	Somatic
12418–12425	ND5	Del A	-	-	Y	1 (1.52%)	0 (0.00%)	Somatic
12540	ND5	A–G	Trp -> Trp	100%	Y	1 (1.52%)	0 (0.00%)	Germline
12794	ND5	T–A	No: Leu -> Ter	100%	N	1 (1.52%)	0 (0.00%)	Somatic
12858	ND5	Ins T	-	-	N	1 (1.52%)	0 (0.00%)	Somatic
12943	ND5	C–T	No: Leu -> Phe	36.6%	N	1 (1.52%)	0 (0.00%)	Somatic

Table S1. *Cont.*

Position	Gene	Change	Amino-Acid Change or Watson-Crick Base-Pairing	Conservation Index (%) [a]	Reported [b]	Number of 66 PTC Patients (%)	Number of 376 Healthy Controls (%)	Somatic/Germline
**Protein-coding region**								
14178	ND6	T–C	No: Ile -> Val	82.9%	Y	3 (4.5%)	3 (0.8%)	Somatic
14310	ND6	C–A	No: Gly -> Trp	70.7%	N	1 (1.52%)	0 (0.00%)	Somatic
14463	ND6	T–C	No: Thr -> Ala	90.2%	Y	1 (1.52%)	0 (0.00%)	Somatic
14518	ND6	A–G	Gly -> Gly	100%	Y	2 (3.03%)	0 (0.00%)	Somatic/Germline
14569	ND6	G–A	Ser -> Ser	100%	Y	2 (3.03%)	14 (3.72%)	Somatic/Germline
14587	ND6	A–G	Gly -> Gly	100%	Y	4 (6.1%)	3 (0.8%)	Somatic/Germline
14668	ND6	C–T	Met -> Met	36.6%	Y	12 (18.18%)	59 (15.69%)	Somatic/Germline
14774	Cytb	C–A	No: Leu -> Ile	63.4%	N	1 (1.52%)	0 (0.00%)	Somatic
14783	Cytb	T–C	Leu -> Leu	26.8%	Y	28 (42.42%)	165 (43.88%)	Somatic/Germline
15018	Cytb	T–A	No: Phe -> Tyr	100%	N	1 (1.52%)	0 (0.00%)	Somatic
**D-Loop region**								
16108	D-Loop	C–T	–	–	Y	3 (4.5%)	2 (0.53%)	Somatic/Germline
16162	D-Loop	A–G	–	–	Y	3 (4.5%)	12 (3.19%)	Somatic/Germline
16164	D-Loop	A–G	–	–	Y	4 (6.1%)	6 (1.6%)	Somatic/Germline
16166	D-Loop	A–G	–	–	Y	1 (1.52%)	2 (0.53%)	Somatic
16172	D-Loop	T–C	–	–	Y	7 (10.6%)	34 (9.04%)	Somatic/Germline
16182	D-Loop	A–C	–	–	Y	8 (12.12%)	38 (10.11%)	Somatic/Germline
16183	D-Loop	A–C	–	–	Y	16 (24.24%)	87 (23.14%)	Somatic/Germline
16184	D-Loop	C–T	–	–	Y	2 (3.03%)	6 (1.6%)	Somatic
16185	D-Loop	C–T	–	–	Y	2 (3.03%)	10 (2.66%)	Somatic
16189	D-Loop	T–C	–	–	Y	21 (31.81%)	112 (29.79%)	Somatic/Germline
16223	D-Loop	C–T	–	–	Y	31 (46.96%)	183 (48.67%)	Somatic/Germline
16259	D-Loop	C–T	–	–	Y	2 (3.03%)	1 (0.27%)	Somatic
16304	D-Loop	T–C	–	–	Y	7 (10.6%)	47 (12.5%)	Somatic/Germline
16362	D-Loop	T–C	–	–	Y	3 (4.5%)	114 (30.32%)	Somatic/Germline
16390	D-Loop	G–A	–	–	Y	1 (1.52%)	3 (0.8%)	Somatic
16482	D-loop	A–G	–	–	Y	1 (1.52%)	0 (0.00%)	Somatic
16519	D-Loop	T–C	–	–	Y	3 (4.5%)	146 (38.83%)	Germline

[a] Conservation index denotes the conservative properties of amino-acid or nucleotides in 41 primate species; [b] According to Mitomap (http://www.mitomap.org); [c] Ter: Terminator.

Table S2. Potentially pathogenic mtDNA variations in RNA region.

Position	Gene	Change	Watson-Crick Base-Pairing [a]	Conservation Index (%) [b]	Reported [c]	Number of 66 PTC Patients (%)	Number of 16 Normal Thyroid Tissues (%)	Number of 376 Healthy Controls (%)	Disease-Associated Mutations Reported in Mitomap
654	12S rRNA	T–C	G–C ↑	90.20%	Y	1 (1.52%)	0 (0.00%)	0 (0.00%)	
2274	16S rRNA	A–G		100.00%	N	1 (1.52%)	0 (0.00%)	0 (0.00%)	
3183	16S rRNA	T–C		80.50%	N	1 (1.52%)	0 (0.00%)	0 (0.00%)	
3244	$tRNA^{Leu(UUR)}$	G–A		100.00%	Y	1 (1.52%)	0 (0.00%)	0 (0.00%)	MELAS, elderly brain,gastric carcinoma, lung tumor, thyroid oncocytoma
3275–3276	$tRNA^{Leu(UUR)}$	Del CA		-	N	1 (1.52%)	0 (0.00%)	0 (0.00%)	
4272	$tRNA^{Ile}$	T–C	A–U ↓	100.00%	N	1 (1.52%)	0 (0.00%)	0 (0.00%)	
4417	$tRNA^{Met}$	A–G		97.60%	Y	1 (1.52%)	0 (0.00%)	0 (0.00%)	
5514	$tRNA^{Trp}$	A–G	A–U ↓	93.75%	Y	1 (1.52%)	0 (0.00%)	2 (0.53%)	Neonatal onset mito disease
5628	$tRNA^{Ala}$	T–C	A–U ↓	87.50%	Y	1 (1.52%)	0 (0.00%)	1 (0.27%)	CPEO/DEAF enhancer
5814	$tRNA^{Cys}$	T–C		85.40%	Y	1 (1.52%)	0 (0.00%)	0 (0.00%)	Mitochondrial Encephalopathy
5835	$tRNA^{Tyr}$	Ins T		-	N	1 (1.52%)	0 (0.00%)	0 (0.00%)	
5881	$tRNA^{Tyr}$	G–C	C–G ↓	100.00%	N	1 (1.52%)	0 (0.00%)	0 (0.00%)	
10463	$tRNA^{Arg}$	T–C		92.70%	Y	1 (1.52%)	0 (0.00%)	0 (0.00%)	Endometrium tumor

[a] Watson-Crick base-pairing: created (↑) or abolished (↓); [b] Conservation index denotes the conservative properties of amino-acid or nucleotides in 41 primate species; [c] According to Mitomap (http://www.mitomap.org).

Table S3. Clinicopathological significance of pathogenic mtDNA mutations in the protein coding region.

Variable	Pathogenic mtDNA Mutation		
	Yes	No	*p* Value
**Age (year)**			
<45	10	20	0.018 *
>45	22	13	
**Sex**			
Male	8	5	0.321
Female	24	28	
**Micro-PTC [a]**			
No	28	24	0.137
Yes	4	9	
**Bilaterality**			
Unilateral	20	19	0.685
Bilateral	12	14	
**Multifocality**			
Single	18	16	0.531
Multiple	14	17	
**With Hashimoto thyroiditis [b]**			
No	27	28	0.796
Yes	4	5	
**Adenoma [b]**			
No	30	30	0.333
Yes	1	3	
**Extrathyroid invasion**			
No	27	30	0.423
Yes	5	3	
**Lymph node metastasis**			
No	19	18	0.694
Yes	13	15	
**Tumor stage**			
I–II	19	28	0.022 *
III–IV	13	5	
**Recurrence [b]**			
No	20	18	0.508
Yes	5	7	
**Novel mtDNA variation**			
No	11	26	<0.001 *
Yes	21	7	
**Heteroplasmic mtDNA variation**			
No	11	20	0.034 *
Yes	21	13	
**Somatic mtDNA variation**			
No	12	25	0.002 *
Yes	20	8	

* *p* values < 0.05 were considered as statistically significant; [a] micro-PTC, papillary thyroid cancer in which the size of all foci is <1 cm; [b] In some patients, the information was unknown.

Table S4. Influence of mtDNA haplotypes in thyroid cancer occurrence.

Haplotype	PTC Group (*n* = 66)	Control Group (*n* = 369) [a]	OR (95% CI)	*p* Value
M	30	184	0.8379 (0.4952–1.418)	0.509
D	16	81	1.138 (0.6153–2.104)	0.68
D4	11	60	1.03 (0.5094–2.083)	0.934
D4a	4	14	1.636 (0.5212–5.134)	0.395
D4b	3	11	1.55 (0.4204–5.713)	0.507
D5	5	20	1.43 (0.5172–3.956)	0.488
G	1	18	0.3 (0.03934–2.288)	0.218
M7	5	25	1.128 (0.4156–3.061)	0.813
M8	7	37	1.065 (0.4531–2.501)	0.886
M74a	1	0	-	0.018
N	36	185	1.194 (0.7054–2.019)	0.509
A	6	25	1.376 (0.5416–3.496)	0.501
A4	4	6	3.903 (1.070–14.23)	0.027 *
N9	4	31	0.7034 (0.2398–2.064)	0.52
N9a	2	26	0.4123 (0.09544–1.781)	0.221
Y	3	5	3.467 (0.8079–14.87)	0.076
R	26	129	1.209 (0.7060–2.072)	0.488
B	12	70	0.9492 (0.4820–1.869)	0.88
B4	11	43	1.516 (0.7370–3.120)	0.255
B4a	4	6	3.903 (1.070–14.23)	0.027 *
B4b	5	13	2.245 (0.7724–6.523)	0.128
B4g	2	1	11.5 (1.027–128.8)	0.013 *
B5	1	22	0.2427 (0.03213–1.833)	0.137
F	13	59	1.289 (0.6611–2.513)	0.455

[a] In some patients, the information was unknown; * *p* values <0.05 were considered as statistically significant.

Table S5. Influence of mtSNPs in thyroid cancer occurrence.

Position	Gene	Change	Amino-Acid Change or Watson-Crick Base-Pairing [a]	Conservation Index (%) [b]	PTC Group (*n* = 66)	Control Group (*n* = 376)	OR (95% CI)	Frequency in 16 Normal Thyroid Tissues (%)
16164 [c]	D-Loop	A–G			4	6	3.98 (1.091 to 14.51)	0.00%
16217	D-Loop	T–C			11	22	3.22 (1.479 to 7.004)	12.50%
16266	D-Loop	C–T			4	6	3.98 (1.091 to 14.51)	0.00%
16362	D-Loop	T–C			10	114	0.41 (0.2022 to 0.8331)	43.75%
16519	D-Loop	T–C			35	146	1.78 (1.051 to 3.010)	43.75%
195	D-Loop	T–C			10	18	3.55 (1.560 to 8.088)	12.50%
263	D-Loop	A–G			65	303	15.66 (2.137 to 114.8)	87.50%
489	D-Loop	T–C			30	116	1.87 (1.097 to 3.179)	43.75%
499	D-Loop	G–A			7	7	6.25 (2.117 to 18.48)	6.25%
709	12s RNA	G–A	G-C ↓	58.50%	5	67	0.38 (0.1463 to 0.9769)	31.25%
5460	ND2	G–A	No: Ala -> Thr	4.90%	6	11	3.32 (1.183 to 9.311)	0.00%
6680	COI	T–C	Thr -> Thr	85.40%	6	9	4.08 (1.401 to 11.87)	0.00%
9123	ATPase6	G–A	Leu -> Leu	97.60%	4	6	3.98 (1.091 to 14.51)	0.00%
14587	ND6	A–G	Gly -> Gly	100.00%	4	5	4.79 (1.251 to 18.32)	0.00%
15043	Cytb	G–A	Gly -> Gly	100.00%	30	91	2.61 (1.522 to 4.474)	43.75%

[a] Watson-Crick base-pairing: abolished (↓); [b] Conservation index denotes the conservative properties of amino-acid or nucleotides in 41 primate species; [c] The mtSNPs had the influence in the predisposition of thyroid cancer were highlighted by bold and italic.

Table S6. mtDNA sequence of 41 vertebrate species.

No.	Species Name	Genbank Accession Number
1	*Cebus albifrons*	NC_002763.1
2	*Chlorocebus aethiops*	NC_007009.1
3	*Chlorocebus pygerythrus*	NC_009747.1
4	*Chlorocebus sabaeus*	NC_008066.1
5	*Chlorocebus tantalus*	NC_009748.1
6	*Colobus guereza*	NC_006901.1
7	*Daubentonia madagascariensis*	NC_010299.1
8	*Eulemur fulvus fulvus*	NC_012766.1
9	*Eulemur fulvus mayottensis*	NC_012769.1
10	*Eulemur macaco macaco*	NC_012771.1
11	*Eulemur mongoz*	NC_010300.1
12	*Galago senegalensis*	NC_012761.1
13	*Gorilla gorilla*	NC_001645.1
14	*Gorilla gorilla gorilla*	NC_011120.1
15	*Homo sapiens*	NC_012920.1
16	*Homo sapiens neanderthalensis*	NC_011137.1
17	*Hylobates lar*	NC_002082.1
18	*Lemur catta*	NC_004025.1
19	*Loris tardigradus*	NC_012763.1
20	*Macaca mulatta*	NC_005943.1
21	*Macaca sylvanus*	NC_002764.1
22	*Nasalis larvatus*	NC_008216.1
23	*Nycticebus coucang*	NC_002765.1
24	*Otolemur crassicaudatus*	NC_012762.1
25	*Pan paniscus*	NC_001644.1
26	*Pan troglodytes*	NC_001643.1
27	*Papio hamadryas*	NC_001992.1
28	*Perodicticus potto*	NC_012764.1
29	*Piliocolobus badius*	NC_008219.1
30	*Pongo abelii*	NC_002083.1
31	*Pongo pygmaeus*	NC_001646.1
32	*Presbytis melalophos*	NC_008217.1
33	*Propithecus coquereli*	NC_011053.1
34	*Pygathrix nemaeus*	NC_008220.1
35	*Rhinopithecus roxellana*	NC_008218.1
36	*Saimiri sciureus*	NC_012775.1
37	*Semnopithecus entellus*	NC_008215.1
38	*Tarsius bancanus*	NC_002811.1
39	*Tarsius syrichta*	NC_012774.1
40	*Trachypithecus obscurus*	NC_006900.1
41	*Varecia variegata variegata*	NC_012773.1

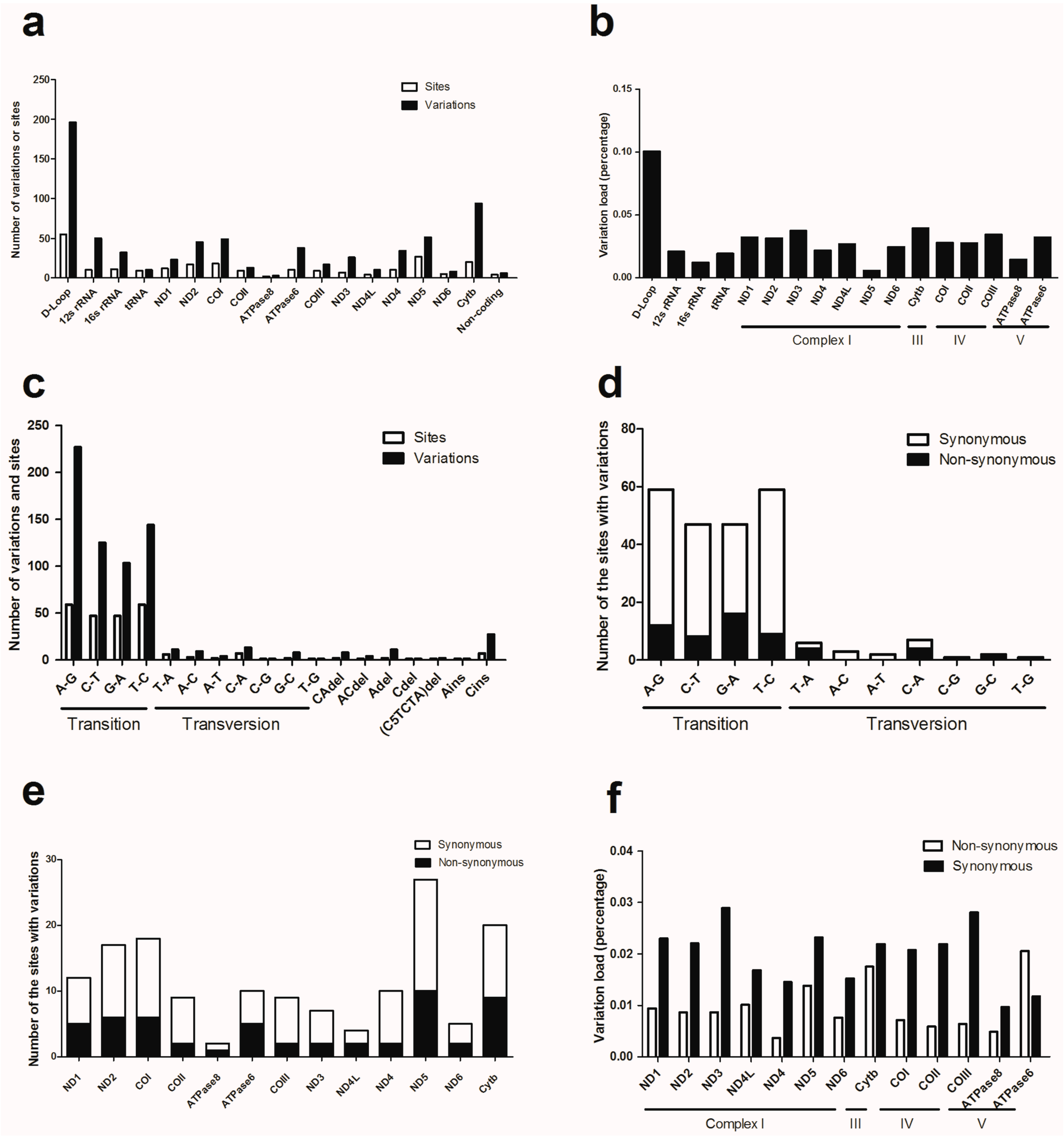

Figure S1. Distribution of mtDNA variations in PTC. (**a**) Distribution of mtDNA variations in the entire mitochondrial genome; (**b**) Variation load in genes and complexes; (**c**) Distribution of different types of variation; (**d**) The ratio of synonymous and nonsynonymous variations in different types of substitution; (**e**) The ratio of synonymous and nonsynonymous variations in genes of protein-coding region; (**f**) Variation load of synonymous and nonsynonymous variations in genes and complexes.
